# Interventions designed to improve the quality and efficiency of medication use in managed care: A critical review of the literature – 2001–2007

**DOI:** 10.1186/1472-6963-8-75

**Published:** 2008-04-07

**Authors:** Christine Y Lu, Dennis Ross-Degnan, Stephen B Soumerai, Sallie-Anne Pearson

**Affiliations:** 1 Department of Ambulatory Care and Prevention, Harvard Medical School and Harvard Pilgrim Health Care, Boston, MA USA; 2 Faculty of Medicine, University of New South Wales and Clinical School, Prince of Wales Hospital, Sydney, Australia

## Abstract

**Background:**

Managed care organizations use a variety of strategies to reduce the cost and improve the quality of medication use. The effectiveness of such policies is not well understood. The objective of this research was to update a previous systematic review of interventions, published between 1966 and 2001, to improve the quality and efficiency of medication use in the US managed care setting.

**Methods:**

We searched MEDLINE and EMBASE for publications from July 2001 to January 2007 describing interventions targeting drug use conducted in the US managed care setting. We categorized studies by intervention type and adequacy of research design using commonly accepted criteria. We summarized the outcomes of well-controlled strategies and documented the significance and magnitude of effects for key study outcomes.

**Results:**

We identified 164 papers published during the six-year period. Predominant strategies were: educational interventions (n = 20, including dissemination of educational materials, and group or one-to-one educational outreach); monitoring and feedback (n = 22, including audit/feedback and computerized monitoring); formulary interventions (n = 66, including tiered formulary and patient copayment); collaborative care involving pharmacists (n = 15); and disease management with pharmacotherapy as a primary focus (n = 41, including care for depression, asthma, and peptic ulcer disease). Overall, 51 studies met minimum criteria for methodological adequacy. Effective interventions included one-to-one academic detailing, computerized alerts and reminders, pharmacist-led collaborative care, and multifaceted disease management. Further, changes in formulary tier-design and related increases in copayments were associated with reductions in medication use and increased out-of-pocket spending by patients. The dissemination of educational materials alone had little or no impact, while the impact of group education was inconclusive.

**Conclusion:**

There is good evidence for the effectiveness of several strategies in changing drug use in the managed care environment. However, little is known about the cost-effectiveness of these interventions. Computerized alerts showed promise in improving short-term outcomes but little is known about longer-term outcomes. Few well-designed, published studies have assessed the potential negative clinical effects of formulary-related interventions despite their widespread use. However, some evidence suggests increases in cost sharing reduce access to essential medicines for chronic illness.

## Background

Managed care organizations (MCOs) are the predominant form of health insurance coverage in the United States. MCOs provide health care to over 160 million enrollees and almost 90% of physicians have at least one managed care contract [1]. By enrollment numbers in 2005, preferred provider organizations (PPOs) are the most common form of managed care (61%) followed by health maintenance organizations (HMOs; 21%), and point-of-service and conventional plans cover a small portion of the insured (15% and 3%, respectively) [1].

Healthcare payers, including MCOs, grapple with the challenge of providing access to essential care that improves health outcomes in the face of increasing need to control healthcare costs. Growth in pharmaceutical spending over the past decade is partly due to increased ingredient costs per prescription, higher levels of utilization, and changes in the drugs being prescribed [2]. A variety of strategies have been used by MCOs to contain escalating drug expenditures and to improve the quality of medication use.

Drug formularies are a common cost containment strategy used by healthcare payers. Formularies are lists of preferred pharmaceutical products covered by an institution within various therapeutic categories [3]. Health plans continue to switch from 1-tier plans (same copayment for all medications under coverage) and 2-tier plans (a lower copayment for generic drugs and a higher copayment for brand-name drugs) to 3-tier plans that include a third, higher copayment(s) for non-preferred brand-name medications. Such 3-tier plans are now the dominant managed care formulary structure [4]. Since 2004, a number of plans have created a fourth tier of cost-sharing for specific types of drugs such as lifestyle medications and biologics [5]. Patient copayments for prescription drugs are another strategy which aims to sensitize patients to the costs of medications so as to discourage use of non-essential medications. Copayments in health plans have risen substantially over the past decade [5]. From 2000 to 2006, the average copayment for generic drugs increased 57% (from $7 to $11), while copayments for preferred brand-name drugs increased 85% (from $13 to $24) and copayments for non-preferred brand-name drugs increased 123% (from $17 to $38) [5]. In addition, MCOs attempt to influence medicines use and improve quality of care through educational programs, prescribing feedback, and computer-based information system [6]. Disease management programs are also used to improve care delivery and health outcomes for patients with specific chronic illnesses [6].

The validity and reliability of information about the effectiveness and unintended consequences of drug-related interventions depends heavily on the strength of the research design [7,8]. By critically appraising studies, systematic reviews can provide useful and more objective information to clinicians, patients, and policy makers. In our previous systematic review of studies evaluating drug-related interventions in MCOs published prior to June 2001 [6], we found a number of consistently effective interventions. These included participatory clinical guideline development, one-to-one and group educational outreach, and enhanced patient-specific feedback. Dissemination of educational materials alone and aggregate feedback to clinicians about their patients were shown to be ineffective. These findings are consistent with other reviews evaluating interventions outside the managed care setting [7,9,10]. We also found that dissemination of educational materials with drug samples – a technique used widely by the pharmaceutical industry to influence prescribing and patient demand – was effective in changing medication use, while disease management showed promise in improving short-term outcomes in the management of diabetes and depression [6]. Importantly, methodologically acceptable studies of the impact of formulary changes and financial strategies as drug cost-containment policies were too few to be included in our previous review. This represented a significant gap in knowledge because such policies are widely used to control pharmaceutical expenditures and it is likely they have negative as well as positive impacts on clinical outcomes. At that time there was also a paucity of evidence of the effectiveness of interventions in more 'lightly' managed care.

The purpose of this review is to update our previous systematic review of interventions to improve medication use in MCOs [6]. Specifically, we aim to describe interventions to improve the quality and efficiency of medication use in the US managed care setting published between July 2001 and January 2007; detail key features of methodologically adequate studies; summarize intervention effects; and identify the most successful types of interventions.

## Methods

### Search Strategy

We performed a systematic review of published interventions conducted in MCOs to reduce pharmaceutical costs or improve use of medicines. We searched MEDLINE and EMBASE from 2001 through January 2007 using a combination of search terms describing the study setting (e.g. managed care programs, health maintenance organizations, preferred provider organizations), methodology (e.g. randomized controlled trials, intervention studies, program evaluation, health services research, comparative studies), intervention types (e.g. formulary, education, practice guidelines, cost containment, quality assurance, risk sharing, reimbursement mechanisms), drug use (e.g. prescription and non-prescription drugs, drug therapy, drug utilization, drug monitoring, drug substitution), and clinician practice patterns (e.g. physician, clinicians, provider, practitioner, physician practice patterns, prescribing practice). The reference lists of studies identified in this way were reviewed to identify studies that our search strategy may have missed. The search retrieved all studies published since January 2001 including some of those analyzed in our previous review.

A total of 2899 articles were identified by the search. Two investigators (CYL, SAP) each independently reviewed half of the study abstracts. Studies were included in the review if they met the following criteria: were published between July 2001 and January 2007 (inclusive), conducted in the US managed care setting including all forms of HMOs, PPOs, and independent practice associations (IPAs); described intervention(s) targeting medication use, including over-the-counter medications, herbals, or vitamins; included a clear description of methods; and measured drug-related outcomes. Disease management interventions focusing on pharmacotherapy were also considered. We have also included in the current review the four methodologically adequate formulary interventions not reported in our previous review. Due to the rapid growth in research on this topic since 2001, there are now sufficient studies to draw more valid conclusions. Clinical effectiveness trials and cost effectiveness studies of medications, descriptive studies, and those examining vaccinations were excluded.

### Rating Study Quality

We reviewed the methodological adequacy of studies identified by our search. Based on the ability of specific research designs to control adequately for common threats to internal validity [8,11,12], our definition for methodologically adequate studies included: randomized controlled trials (RCTs); pre-post studies with non-randomized comparison group(s); and interrupted time series analysis with or without comparison group. Interrupted time series designs examine changes in outcomes of interest using multiple observations (at least 4 by our definition) before and after an intervention [12]. Research designs deemed methodologically inadequate were pre-post studies without a comparison group, non-randomized post-only studies, and cross-sectional studies. Such designs have been shown to produce invalid results in previous research [7,8]. In addition, studies were only included if each comparison group included at least 20 subjects to ensure the reliability of results.

### Data extraction

We extracted key features of the characteristics, methods, and outcomes of methodologically adequate studies using a standardized data extraction tool. Key features included: setting, objective/s, health condition/s addressed, intervention components, predominant intervention strategy, intervention target/s, and up to three primary outcome measures reported by the authors. To check reliability of data extraction, two investigators (CYL, SAP) independently extracted data from the included studies and calculated effect sizes of outcome measures for one-third of the studies in this review.

## Results

One hundred and fifty one published studies met our initial inclusion criteria, of which 31% met our criteria for minimally acceptable methodology. The majority of these studies (45%) were conducted in staff, group and mixed model settings and 6 studies were conducted in IPAs, network model organizations, and Medicare or Medicaid MCOs; the remaining studies (40%) did not provide detailed information on the type of managed care setting.

We classified studies according to predominant strategy and key intervention components [Table 1]. In order of frequency, predominant strategies were formulary-related interventions (40% of studies), disease management (25%), monitoring and feedback (13%), educational interventions (12%), and collaborative care involving pharmacists (10%).

**Table 1:** Studies identified according to key features and predominant intervention strategy

	**Educational**	**Monitoring & Feedback**	Formulary*	**Collaborative Care Involving Pharmacists**	**Disease Management**†	**TOTAL**
**All studies**	20	22	66	15	41	164
**Methodologically acceptable studies**	7	16	15	5	8	51
RCT	5	10	2	3	7	27
Interrupted time series	0	5	5	0	0	10
Pre/post studies with comparison	2	1	8	2	1	14
**Interventions in methodologically acceptable studies**‡	17	18	21	5	8	69

* published studies from 1966 through January 2007.

† Only studies with pharmacotherapy as a primary focus.

‡ Studies may have examined more than one intervention and multiple studies may have examined the same intervention

The primary interventions tested in studies that met criteria for methodological adequacy were: educational interventions (7), monitoring and feedback (16), formulary-related interventions (15 including 4 published prior to July 2001), collaborative care involving pharmacists (5), and disease management (8). We categorized methodologically adequate interventions involving financial incentives as formulary-related interventions because the predominant focus of these studies was to assess the effects of differential patient copayment associated with tiered formularies. Additional file 1 summarizes the key aspects of the 51 methodologically acceptable studies.

### Educational interventions

#### i. Dissemination of Educational Materials Alone

The dissemination of educational materials alone (e.g. newsletters, clinical guidelines, and audiovisual materials) is used commonly as a behavior change strategy, although this approach has been shown repeatedly to be ineffective as a stand-alone intervention [6,9]. This strategy was one arm of an RCT conducted by Majumdar et al [13] in the form of a clinical guideline and 'toolkit' (a list of eligible patients and patient educational materials); another arm of this trial evaluated the impact of group education (discussed below). Not surprisingly, the materials only intervention failed to increase the testing and treatment of Helicobacter pylori (HP) infection or to reduce proton pump inhibitor and histamine-2 blocker prescribing.

#### ii. One-to-One Education

One-to-one educational outreach (often referred to as academic detailing) has been shown to be an effective technique to change many aspects of physician behavior, particularly medication use [14,15]. Simon et al [16] demonstrated that one-to-one education sessions delivered by peer leaders increased guideline adherence and the use of diuretics and beta-blockers for hypertension.

Two studies evaluated the impact of one-to-one patient counseling by pharmacists. One intervention successfully decreased mortality rates in patients using drugs with a narrow therapeutic index; hospitalizations were also reduced by both brief and detailed pharmacist counseling, although the effects were not significant [17]. Results from another study [18] showed that detailed pharmacist counseling on adherence to medications with follow-up telephone calls did not increase the rate of eradication of HP infection over pharmaceutical treatment without counseling, but it did increase patient satisfaction.

#### iii. Group Education

Group education relies on either didactic or problem-based approaches to influence behavior [7]. In our review peer leader education in small practice-based groups was shown to increase testing and treatment for HP infection; however, it was unsuccessful in reducing the use of proton pump inhibitors or histamine-2 blockers [13]. Simon et al [16] demonstrated that an intervention involving group academic detailing improved antihypertensive prescribing; individual academic detailing was more successful than group visits in increasing the use of diuretics and beta-blockers as recommended by clinical guidelines. There is some evidence that both group and individual detailing interventions are cost-saving [16,19]. Another study using group academic detailing by psychiatrists and interactive discussions to improve depression management by primary care physicians demonstrated no significant increase in antidepressant prescribing or treatment duration at 12 months post-intervention [20]. A significant reduction in antibiotic prescription rates was achieved over a 3-year period by using a multifaceted intervention comprised of peer leader education sessions to practitioners, provision of mailed educational materials (based on materials from the Centers for Disease Control and Prevention) to parents of young children, and dissemination of key educational messages via radio, newspaper, television and pamphlets in hospitals, clinics, and pharmacies directed at the general public [21].

### Monitoring and feedback

#### i. Prescribing audit and feedback

Audit and feedback interventions report physicians' past or current clinical practice, usually in relation to the practice of peers or accepted standards. Audit and feedback can achieve small to moderate improvements in physicians' practice [22,23]. Four studies in this review used prescriber feedback combined with educational materials to change medication use. Providing clinical recommendations to physicians with annual mailed peer-comparison feedback over a four-year period achieved small increases in the use of statins, beta-blockers, and angiotensin-converting enzyme inhibitors among patients with coronary heart disease [24]. Simon et al [25] demonstrated that internet-based feedback to residents on their prescribing practices complemented by educational materials did not increase the proportion of diabetic patients receiving glycemic monitoring or change antihypertensive prescribing. However, residents were required to actively access a password-protected personalized website to be exposed to the intervention [25].

Two studies targeted physicians and patients using educational materials and feedback. The first achieved a small reduction in antibiotic prescribing for acute respiratory tract infections by delivering practice guidelines and performance feedback to physicians, combined with mailing educational materials to patient households and placing information in waiting and examination rooms [26]. In the second study, a significant increase in antidepressant adherence was achieved by providing physicians with a list of non-adherent patients, guidelines, and feedback on their prescribing, along with mailing educational information to patients initiating therapy and reminder letters to non-adherent patients [27].

#### ii. Computerized real-time alerts

The common types of information technology used in clinical care include electronic medical records, computerized physician order entry, and real-time decision support (such as reminders and prompts). Computerized order entry with clinical decision support has been demonstrated to improve prescribing and reduce medication error rates [28]. Smith et al [29] used a computerized provider order entry system to provide prescribing alerts regarding medicines with potential contraindications and therapeutic alternatives. They found a reduction in dispensing rates of potentially contraindicated medicines (including tricyclic-antidepressants) among the elderly (aged > 65 years). Another intervention of electronic reminders to providers successfully increased the proportion of female patients receiving osteoporosis medications or bone mineral density measurements; total calcium intake, as a measure for quality of care, also increased [30]. The addition of patient reminder letters did not increase the impact of alerts alone on the target outcomes [30].

Two studies in this review combined computerized alerts and group academic detailing [31,32]. Interrupted time series analyses demonstrated that computer-based drug-specific and patient age-specific alerts were effective in reducing prescribing of medications that interact with warfarin [31] and the use of medications contraindicated in the elderly [32]. In both studies, group education failed to achieve any additional change in medication use over the alerts alone.

#### iii. Reminders and telephone outreach

Bambauer et al [33] evaluated the impact of alerting prescribers via faxed letters about patients who had gaps of more than 10 days in refilling anti-depressant prescriptions during the first six months of therapy. The faxed alerts to prescribers had no discernable effect on the proportion of non-adherent patients or the number of days without antidepressant treatment during the 12-month follow-up period.

The effectiveness of computerized reminders has also been demonstrated to improve rates of recommended laboratory drug monitoring. In two studies by Raebel et al [34,35] daily computerized alerts identifying incomplete laboratory tests for a range of drugs (such as amiodarone, carbamazepine, and statins) were sent to pharmacists who telephoned patients about the missing tests. This intervention successfully increased the completion of laboratory monitoring both at the initiation of and during the course of drug therapy [34,35]. In contrast, neither non-intrusive computerized alerts to physicians, nor presenting information on computer screens without requiring additional actions, were ineffective in changing laboratory monitoring rates at the initiation of drug therapy [36].

A study by Feldstein et al [37] compared three interventions to increase laboratory test completion for drugs prescribed frequently in primary care. Electronic reminders to providers about missing laboratory tests, with accompanying sample letters for distribution to patients, were successful in increasing the proportion of patients completing tests; however, the impact was greater when patients were contacted directly. Two other interventions – reminding patients about incomplete tests via automated telephone voice message or telephone calls by nurses – were both more effective than patient-specific alerts to providers in increasing laboratory test completion [37].

Telephone outreach to patients by pharmacists was evaluated in three papers. In one study [38,39] three monthly telephone calls by pharmacists to monitor patients taking antidepressants increased medication adherence and improved depression symptoms, and not surprisingly, increased patient feedback to pharmacists about medication use [38,39]. However, in the management of asthma, three outreach telephone calls about five months apart with tailored feedback to patients did not change medication or healthcare utilization [40].

### Formulary interventions

#### i. Tiered formularies

Tiered formularies with different levels of patient copayment to encourage use of preferred drugs are used increasingly to enhance cost-effective use of medicines [41]. Six papers in this review evaluated the impact of tiered formulary changes.

Changing from a single copayment to a three-tier structure reduced continuation of the original medications, increased switching to agents in a lower tier, discontinued use of all drugs in the affected class in some patients, and decreased plan spending while increasing enrollee spending [42,43]. Similar but smaller effects were observed when changing from a two-tier to a three-tier formulary [42]. These changes in the pattern of medication use were seen in a number of prescription drug classes examined, including angiotensin-converting enzyme inhibitors, statins, proton pump inhibitors, and medications for attention-deficit hyperactivity disorder [42,43]. A change from two to three tiers decreased claims for third tier medications and net insurer costs, while patient out-of-pocket copayments increased significantly in both the first year [44] and second year [45] of follow-up. In a study by Nair et al [46] change from a two-tier to a three-tier design increased compliance of prescribers to preferred medications and increased overall medication use (primarily due to increases in use of medications in the two lower tiers).

Patient cost-sharing (either in the form of fixed, percent, or tiered copayments) is an important factor influencing medication use. Roblin et al [47] showed that large (> $10 per 30-day supply) and moderate ($7–$10) increases in cost-sharing attenuated an increasing trend of oral hypoglycemic medication use by 18.5% and 9.2% respectively over a 6-month period, while small ($1–$6) increases in copayments had no impact.

#### ii. Prior authorization

Prior authorization (PA) policies are commonly used to control drug utilization and cost [48]. In a health plan that instituted a PA policy for COX-2 inhibitors, Hartung et al [49] showed a significant reduction in COX-2 inhibitor use as well as a small decrease in use of gastrointestinal protectants (e.g. proton pump inhibitors and histamine-2 blockers) at one year post-intervention. Another study demonstrated a reduction in pharmacy utilization but an increase in medical utilization (particularly hospitalization and emergency department visits) after a COX-2 inhibitor PA policy was implemented [50].

#### iii. Formulary/coverage change

After vaginal anti-fungal products became available over-the-counter, Gurwitz et al [51] showed a significant reduction in anti-fungal prescriptions. Studies by McDonough et al [52] and Andrade et al [53] both evaluated the impact of formulary switches between agents in the same therapeutic class and found increases in the prescribing of the preferred agents (lisinopril and esterified estrogen tablets, respectively). After loratidine became available as an over-the-counter medication, Sullivan et al [54] found that prescriptions decreased for both antihistamines and the total category of allergic rhinitis medications (including inhaled corticosteroids and leukotriene receptor antagonists) with a substantial reduction in pharmacy costs for these medications. Antihistamine prescriptions were further decreased by switching antihistamines to the third formulary tier or by imposing a PA policy for these medications [54].

Two studies by Delate et al [55,56] evaluated the effects of a formulary change with communication to physicians and/or patients informing them of the change. The first study evaluated the impact of notifying patients via mail of impending formulary changes [56] and showed an increase in subsequent formulary adherence. The second study used letters to notify physicians and patients of formulary changes and showed a small increase in conversion of inefficient dosage regimens to a recommended daily dosage of the same medication [55]. Similarly, DeZearn et al [57] evaluated the effect of formulary change with letters to prescribers encouraging the use of generic cimetidine and found a reduction in prescriptions for the brand name product and an increase in the prescribing of generic cimetidine.

### Collaborative care involving pharmacists

Collaboration of pharmacists in the care process has emerged as a promising approach to improve quality of care and patient outcomes [58]. Five studies in this review that evaluated collaborative care interventions supported evidence of effectiveness.

Finley et al [59] showed increases in antidepressant adherence and small reductions in resource utilization when pharmacists were used to titrate antidepressant doses, under the supervision of psychiatrists. Okamoto et al [60] demonstrated reduction in blood pressure and improvement in patient quality of life (as measured by SF-36) without increasing total costs per patient when pharmacists were responsible for educating patients, altering therapy by administering more appropriate or less expensive drugs to achieve similar or improved blood pressure control, and ordering laboratory tests as required. Another study showed a significant increase in the proportion of patients receiving screening and a reduction in total cholesterol and low-density lipoprotein cholesterol levels when pharmacists were responsible for monitoring and titrating medications, and monitoring laboratory testing [61]. Straka et al [62] evaluated the impact of collaboration between pharmacists and providers in developing patient-specific care plans which were followed by pharmacists monitoring and educating patients. The intervention was successful in reducing low-density lipoprotein levels over the short-term and the effects persisted 18 months after discontinuation of the intervention; the proportion of patients achieving the low-density lipoprotein cholesterol goal also increased significantly [62]. Borenstein et al [63] evaluated the impact of a multifaceted program involving individual and group education sessions for physicians run by pharmacists, provision of a list of patients with uncontrolled hypertension, individualized patient education, monitoring by pharmacists, and hypertension clinics to assess blood pressure and adherence to antihypertensive medications. Treatment decisions by physicians considered assessment results and blood pressure measures provided by pharmacists. This intervention effectively reduced systolic blood pressure and increased the proportion of patients achieving blood pressure goal level [63]. A non-significant increase in total provider visits (physician and pharmacist visits) was also observed [63].

### Disease management interventions

Disease management programs are structured, population-based approaches for identifying, treating, and monitoring persons with chronic illness. Generally, these are multidisciplinary, integrated approaches aimed to improve processes of care and patient outcomes [64]. Seven papers describing disease management interventions that had pharmacotherapy as a primary focus were included in this review.

#### i. Depression

Depression is a common medical condition that is associated with significant social and functional impairment as well as high direct and indirect health care costs [65]. Research suggests depression can cause greater functional disability than other chronic conditions such as diabetes, chronic lung disease, and hypertension [66].

In one study, psychiatrists reviewed patient psychosocial history and current depressive episodes over multiple sessions. The intervention, which also included books and videotapes for patient education and pharmacologic treatment where necessary, effectively increased antidepressant adherence at 12 months with modest improvements in depression symptoms among severely depressed patients [67]. Katon et al [68] evaluated an individualized stepped-care depression treatment program provided by specialist nurses working collaboratively with primary care physicians for diabetic patients diagnosed with depression. The intervention increased the use of adequate antidepressant doses and drug adherence at 12 months; there were also modest improvements in depression symptoms and no change in diabetes outcomes [68]. Unutzer et al [69] evaluated a quality improvement intervention targeting treatment of depression with follow-up by specialist nurses. The intervention increased the proportion of patients using antidepressants and antidepressant use according to guideline-recommended doses among patients at high risk for relapse. In the same study, an intervention with a focus on quality of psychotherapy rather than medication use was less successful than the intervention targeting improved medication therapy [69].

Ray et al [70] examined the impact of transitioning patients from traditional Medicare and MCOs to specialty behavioral health organizations ('carve-out' programs). They found a significant reduction in the proportion of patients who continued antipsychotic therapy; this negative effect was greatest in high-risk patients.

#### ii. Asthma

Lozano et al [71] evaluated the impact of two interventions: physician peer leader education within practice groups and peer leader education coupled with nurse care planning, telephone monitoring and follow-up of medication use, asthma symptoms and support for self-management. Both interventions effectively reduced asthma symptoms and decreased the use of oral steroids; the planned care intervention (multifaceted) had a greater effect on these target outcomes than the group education intervention [71]. However, at the practice-level, the two intervention approaches did not differ in changes in the proportion of patients using controller medications or oral steroids, or in frequency of medical visits [72].

#### iii. Helicobacter pylori infection

Published guidelines recommend testing for HP in patients receiving long-term acid suppression therapy for acid-related disorders, and antibiotic treatment for those with HP-positive peptic ulcer disease [73,74]. A test and treat intervention for HP infection in patients receiving long-term acid-reducing therapy successfully improved gastrointestinal symptoms and reduced the use of acid-reducing medications and associated costs at 12 months [75]. Ofman et al [76] evaluated another test-and-treat program for HP infection provided collaboratively by physicians, pharmacists and nurses. This multifaceted intervention involved local development and implementation of test-and-treat guidelines, individual and group academic detailing, patient education by pharmacists, and HP testing and medication monitoring by nurses. The intervention successfully increased compliance with HP testing and the proportion of patients prescribed appropriate anti-HP therapy at six months with no significant change in total costs of related health services [76].

## Discussion

There has been a substantial increase in published research evaluating interventions targeting medication use in managed care settings since our previous review [6]. This review identified 151 intervention studies in a six-year publication period using a narrower inclusion criteria (that is, disease management studies only included where pharmacotherapy was a primary focus) compared with 105 studies over 35 years in our previous review (70 of which were reported since 1996). Despite the increase in research on this topic, a smaller proportion of published studies met our criteria for methodological adequacy (31% versus 46%). This failure to increase the degree of methodological rigor in published research is of concern because significant resources are used conducting studies of poor methodological quality and such research may produce scientifically invalid conclusions that influence future research and policy. Although not a focus of this review, we noted additional methodological issues apart from research design. For example, the proxy measures of adherence varied between studies (n = 10). Three studies used the medication possession ratios [20,27,59], while others measured the proportion of patients continuing treatment [33,38,42,43,67,68,70]. The variability in measures of adherence and infrequent reporting of this important outcome in part reflects the well-known methodological challenges in the measurement of adherence [77].

Several findings from this report are consistent with our previous review and the results of other systematic reviews on the impact of interventions designed to change medication use. We once again confirmed that dissemination of educational materials alone is ineffective and one-to-one educational outreach visits are effective in increasing adherence to prescribing guidelines [6,9,10,15,78]. Further, this review confirms that multifaceted interventions are more likely to be successful in changing medication use than those using single strategies [6,10,78].

The current analysis, like other reviews, suggests group education interventions may be beneficial but the findings are inconclusive [78,79]. We found that peer leader group education was effective in changing medication use but was less effective than one-to-one academic detailing [16,31,32]. The absence of incremental impact of group education when combined with computer-based alerts, as reported by Simon et al [32] and Feldstein et al [31], may reflect the weakness of the approach or the superior effectiveness of computerized alerts in changing the particular outcomes targeted in these interventions.

Intervention approaches involving monitoring and feedback have been shown previously to produce small to moderate effects on medication use [6,23,80], and this review confirms these findings. Further, our review also supports the existing literature regarding the short-term effectiveness of real-time computer-based alerts in changing prescribing and test ordering [28,81]. However, most studies in our review evaluated electronic medical record systems with particular characteristics in a single HMO, thus limiting the generalizability of findings. Only one study [30] reported patient-relevant outcomes (namely, quality of life measures).

Our review provides evidence that tiered formulary and patient copayment interventions decrease non-preferred drug use, reduce overall insurer costs, and increase patient out-of-pocket expenses as intended. The use of prior authorization policies resulted in similar effects. However, our findings concur with previous reviews in managed care and other settings that these interventions may also be associated with undesirable effects such as increased rates of switching to other medications or discontinuation of essential and cost-effective medications [3,48,82,83]. In addition, some studies reported changes in measures such as hospitalization or medical resource utilization (e.g. office visits) resulting from formulary-related interventions [44,45,49,50,70].

Evidence from the literature suggests that coordinating pharmacist services as a component of patient care improves quality of care [84-86]. This review documents positive results for collaborative care led by pharmacists, including improved drug adherence and clinical outcomes. Only some data on costs and resource use (e.g. cost per mmHg of blood pressure decrease, average provider visit cost per patient) have been reported [59,60,63].

Finally, our findings confirm that disease management interventions are associated with improvements in the process and quality of care, both short and longer term [87]. However, these disease management interventions are by their nature multifaceted, and the type and number of features within each disease management program vary widely. Therefore, it is difficult to draw firm conclusions about the contribution of individual components to the overall impact.

There are several limitations to this review which need to be acknowledged. Despite our intensive efforts to identify all of the published literature on interventions to improve the quality and efficiency of medication use in US managed care settings, our search strategy may have missed published articles. The full range of interventions currently used by managed care programs to influence use of medicines is not covered by the studies in our review. For example, no studies evaluated the impact of physician incentives on prescribing, despite that fact that this strategy is commonly used by MCOs. Further, our findings are likely to be subject to under-reporting bias with interventions showing no or negative effects, which are less likely to be reported in the peer-reviewed literature. Even when studies are published, many are methodologically inadequate; 69% of the studies identified in this review were not included in the detailed analyses because of design flaws. There are also fundamental difficulties in comparing interventions with diverse objectives, intervention targets, measurement methods, and outcomes. Finally, it is difficult to reach definitive conclusions about the contribution of individual components to the overall effect of multifaceted interventions.

### Future Directions

Despite the substantial number of interventions to improve drug use in managed care, our understanding of the impacts of these interventions is still limited.

First, a large proportion (40%) of the reviewed studies did not detail the specifics of the managed care setting within which the study was conducted. We found few studies conducted in PPOs despite their prominent role in the US managed care industry or in other "lightly" managed care settings. The majority of studies in our review were conducted in staff, group, or mixed-model HMOs, which represent more heavily managed settings. Future research should establish whether intervention approaches that are successful in such settings are also effective in more lightly managed settings. Second, most studies in this review used common surrogate outcome measures, such as prescribing rates or proportions of patients achieving specific medication use practices. Few studies reported medication adherence or patient-relevant outcomes such as clinical status, hospitalization rates, or quality of life measures. Furthermore, evidence about the cost-effectiveness of most interventions is still quite limited. Cost-effectiveness data are needed to select among many possible strategies for improving medication use and associated costs. Third, there is still a lack of publicly available, high quality evidence concerning the effects of interventions involving formulary changes or financial incentives, which are commonly used to influence medication use. Although the increased reporting of formulary-related interventions is encouraging, the high prevalence of inadequate designs (e.g. pre-post without comparison group, post-only) among studies of formulary-related interventions (77%) is striking. An important conclusion to be drawn from this review is that improvements in research methodology will be essential in order to produce valid and reliable study results about cost, quality of care, and patient outcomes to inform managed care policy decisions. It is highly likely these interventions do reduce costs to the payer as they continue to be dominant in the managed care environment. Evaluation of the patient-relevant outcomes of formulary-related interventions is a research priority. Finally, the durability of most of the intervention effects reported in this review is uncertain because few studies extend beyond two years follow-up and many cover even shorter periods. In particular, further research is needed to determine if computerized clinical support systems are associated with improved patient outcomes over the long-term.

## Conclusion

There is good evidence for the effectiveness of several strategies in changing drug use in the US managed care setting. Policy makers have a solid and expanding knowledge base to employ in the design of educational, monitoring and feedback, and collaborative care interventions to improve medication use. Computerized alerts also show promise in improving short-term outcomes. Despite the wide use of formulary-related approaches to managing pharmaceutical use, there are still few well-designed studies assessing their effects on patient outcomes. In order to maximize health care cost-effectiveness while containing costs, it is crucial to continue to promote rigorous testing of interventions to improve medication use in managed care settings in order to expand available high quality evidence about their clinical and economic impacts.

## Competing interests

The author(s) declare that they have no competing interests.

## Authors' contributions

Conception and design: CYL, SAP, DRD, SBS. Administrative support: CYL, SAP. Collection and assembly of data: CYL, SAP. Data analysis and interpretation: CYL, SAP. Manuscript writing: CYL, SAP, DRD, SBS. Final approval of manuscript: CYL, SAP, DRD, SBS.

## Pre-publication history

The pre-publication history for this paper can be accessed here:



## Supplementary Material

Additional file 1 Key features of 51 methodologically acceptable studies Click here for file
