# Supplementary material for: Interventions designed to improve the quality and efficiency of medication use in managed care: A critical review of the literature – 2001–2007
Source: BMC Health Serv Res. 2008 Apr 7;8:75. doi: 10.1186/1472-6963-8-75 (PMC2323373; doi:10.1186/1472-6963-8-75)
Supplement: Additional file 1 — Key features of 51 methodologically acceptable studies [file 1472-6963-8-75-S1.doc]

**Table 2: Key features of 51 methodologically acceptable studies**

| **First author, year** | **Design &**  **Focus** | | **Target Group & Setting** | **Goal/s** | **Intervention/s** | **Key Outcomes** | **Magnitude of Effect (%)^δ^** |
| --- | --- | --- | --- | --- | --- | --- | --- |
| **I. EDUCATIONAL INTERVENTIONS** | | | | | | | |
| Majumdar S 2005 [13] | RCT  Treatment of HP infection | | 581 patients of 14 managed care practices | Increase testing and treatment of HP & reduce chronic use of PPI and H2B. | (I1) passive education: guidelines & accompanying toolkit for the management of this clinical problem. Guidelines to PCPs suggested that a therapeutic reevaluation include testing & treating HP. Toolkit to each PCP include: a list of eligible patients from their practice, patient educational material, "Dear Patient" letters, HP test requisitions, prescription for recommended eradication regimens, & 'progress notes' to add to the chart.  (I2) active education: same guidelines provided + 2 additional methods for guideline dissemination. 'Peer leader' introduced the guidelines and conducted small group academic detailing sessions & pharmacists provided individualized reinforcement of key messages and reminders about eligible patients > 1 month after guideline dissemination. | % Patients received HP testing | (I1) 0  (I2) +20 |
|  |  |  |  |  |  | Increase in rates of HP treatment (incidence rate ratio, IRR) | (I1) IRR=1.45  (I2) IRR=2.96 |
|  |  |  |  |  |  | Reduction in prescription rates of PPI or H2B | (I1) 0  (I2) +0.75 for PPI; 0 for H2B |
| Simon S 2005 [16] | RCT  Hypertension | | 9,820 patients of 9 practices of a mix-model HMO: 2 staff-model & 1 group-model division | Increase diuretic or beta-blocker use in hypertension | (I1) Individual academic detailing: peer leader delivered 15-30 minutes one-to-one session  (I2) Group academic detailing: peer leader delivered 45 minutes small group session, interaction during the sessions was encouraged  (C) mailed educational material describing guidelines for prescribing antihypertensives | Increase in % diuretic or beta-blocker use among new diagnosed patients at 2 years | (I1) +4.6  (I2) +1.2 |
|  |  |  |  |  |  | Switching to diuretic or beta-blocker in prevalent patients treated with other antihypertensives at 2 years | (I1) 0  (I2) 0 |
|  |  |  |  |  |  | Outpatient visits per patient at 1 year | (I1) +25.2  (I2) +9.7 |
| Stevens J 2002 [18] | RCT  Treatment of HP infection | | 302 patients of a non-profit, group-model HMO in the Portland, Oregon | Enhance appropriate use of drug treatment for eradication of HP infection | (I1) 15-minute patient counseling by pharmacists, include a detailed review of possible side effects & a follow-up phone call with the patient 2-3 days after the start of therapy to check on adherence to the drug regimen.  (C) 5-minute counseling | Increase in % patients with HP eradicated at 3 months | (I1) 0 |
|  |  |  |  |  |  | % Patient “very satisfied” with pharmacy services | (I1) +17.4‡ |
| Yuan Y 2003 [17] | RCT  Drug safety | | 5,499 patients of a MCO in Southern California | Increase patient survival & decrease hospitalization | (I1) Pharmacists briefly counsel all patients with new or changed prescription  (I2) Detailed counseling for high-risk patients who use a drug with a narrow therapeutic index  (C) counseling when the patient requested it or the pharmacist determined that counseling was necessary | At 2 years:  Reduction in mortality per new prescription | (I1) 0  (I2) +7.9† |
|  |  |  |  |  |  | Reduction in hospitalization | (I1) +2.9  (I2) +2.3 |
| Simon S 2007 [19] | RCT  Hypertension | | See Simon S 2005 | See Simon S 2005 | See Simon S 2005 | Decrease in average per person daily cost of antihypertensive medication at 2 years | (I1) +$0.0486  (I2) +$0.0597 |
| Lin E 2001 [20] | Random assignment, PP&C  Depression | | 109 PCPs of 15 primary clinics of 2 large HMOs located in the Midwest & Northwest regions | Improve depression management | PCPs received education on diagnosis & optimal management of depression over 3-month training. Methods of education include: small group interactive discussions, expert demonstrations, role-play, & academic detailing of pharmacotherapy. | At 12 months:  Increase in new antidepressant prescriptions /100 visits | -0.26 |
|  |  |  |  |  |  | Increase in new antidepressant prescription for a newly diagnosed episode of depression /100 visits | -0.01 |
|  |  |  |  |  |  | Antidepressant treatment duration | -5 |
| Perz J 2002 [21] | PP&C  Antibiotic prescribing | | Patient number not reported (464,200 person-years). Tennessee Medicaid Managed Care Program in 4 counties | Reduce inappropriate antibiotic use among children. | One-year community wide educational intervention by the County Health Department, primarily based on CDC developed materials. Peer leader education sessions & guidelines distributed to health practitioners. Newsletters & educational materials to parents of young children. Pamphlets in hospitals, clinics, dental offices, pharmacies. Communication through TV, radio, newspaper for public education. Message emphasized: antibiotics should be used for bacterial infection only, colds & most coughs, sore throats more commonly caused by viruses not bacteria, & narrow spectrum antibiotics should be prescribed. | At 3 years:  Reduction in antibiotic prescription rates | +11‡ |
|  |  |  |  |  |  | Reduction in ratio of antibiotic prescriptions to respiratory illness visits | +8 in white; +13 in black |
| **II. MONITORING & FEEDBACK INTERVENTIONS** | | | | | | | |
| **i. Prescribing audit and feedback** | | | | | | | |
| Goff DC  2003 [24] | RCT  Coronary heart disease | | 184 practices (605 physicians) of a network model MCO in northwestern North Carolina | Increase the use of statins, beta-blockers, & ACE inhibitors | Summary of American Heart Association recommendations mailed to practices. Performance audit with peer-comparison performance feedback & patient-specific chart reminders provided. Intervention delivered annually. | At 4 years:  % Patients using statins | +2 |
|  |  |  |  |  |  | % Patients using beta-blockers | -4.8 |
|  |  |  |  |  |  | % Patients using ACE inhibitors | -2.3 |
| Simon S 2005 [25] | ITS  Diabetes  Hypertension | | 964 patients of a 14-sites multi-specialty group in greater Boston | Improve care for diabetes & hypertension by residents. | Residents can access password-protected, personalized website for internet-based audit & feedback on their practice with accompanying educational materials (recommendations, clinical guidelines, & internet links for additional medical information) provided. Resident program directors endorsed this intervention by a letter to each resident. | Increase in diabetic patients with glycemic monitoring/month | 0 |
|  |  |  |  |  |  | Increase in hypertensive patients prescribed a diuretic or beta-blocker | 0 |
| Gonzales R 2004 [26] | RCT  Acute respiratory tract infections | | 55 practices (4,270 patient visits) of a Medicare MCO in Colorado | Improve appropriate antibiotic use for acute respiratory tract infection | Practice guidelines to PCPs. PCPs who provided care to >5 adults with bronchitis during the previous winter period would receive a prescribing profile. Educational materials sent to households, waiting rooms & examination rooms of practices. | Reduction in % total visits resulting in antibiotic prescription | +3 |
|  |  |  |  |  |  | Reduction in % bronchitis visits resulting in antibiotic prescription | +5 |
| Hoffman L 2003 [27] | RCT  Depression | | 9,564 patients of an IPA in Florida | Increase patient adherence to antidepressant medication. | Guidelines provided to physicians with feedback on regional prescribing rates for that practice area, plus patient information & patient letters. Letters to patients. Prescribers received a list of non-adherent patients (missed >10days of therapy) via mail ~20-25 days after the end of each month. Non-adherent patients received a personalized, reminder letter. | At 180 days:  Improvement in antidepressant adherence (MPR) | +0.6‡ |
|  |  |  |  |  |  | Improvement in antidepressant adherence among patients with depression diagnosis (n=1981) | +2.3‡ |
| **ii. Computerized real-time alerts** | | | | | | | |
| Smith D 2006 [29] | ITS  Drug safety | | 209 clinicians of a group-model HMO in the Pacific Northwest | Reduce use of potentially contraindicated agents in elderly persons. | Using a computerized provider order entry system to provide drug-specific alerts for non-preferred medications that carry potential contraindication (e.g. long-acting benzodiazepines & TCAs) in elderly, & provide therapeutic alternatives. | At > 2 years:  Reduction in initial dispensing of non-preferred agents among elderly (/10000) | +22^†^ |
|  |  |  |  |  |  | Reduction in use of non-preferred TCAs for elderly | +35^†^ |
|  |  |  |  |  |  | Dispensing rates of preferred agents | Non-significant effect for elderly; +20^†^ for non-elderly |
| Feldstein A 2006 [30] | RCT  Osteoporosis | | 311 female patients (159 PCPs) of 15 primary care clinics in a Pacific Northwest non-profit, group-model HMO | Increase the proportion of women who received BMD measurement or a medication for osteoporosis after a fracture | (I1) EMR to PCPs which informed the PCP of the patient’s risk of osteoporosis & the need for evaluation and treatment. Follow-up EMR sent again after 3 months.  (I2) EMR to PCP + patient reminder. EMR & advisory letters to patients with educational materials. | Increase in % patients receiving an osteoporosis medication or a BMD measurement at 6 months | (I1) +45.6†  (I2) +37.2† |
|  |  |  |  |  |  | Increase in % patients with regular physical activity | (I1) -11.6  (I2) -6.7 |
|  |  |  |  |  |  | Total calcium intake, mg/day | (I1) +52.5*  (I2) +35.3* |
| Feldstein A 2006 [31] | RCT & ITS  Drug safety | | 239 PCPs at 15 clinics & 9,910 patients of a group-model, non-profit HMO | To reduce warfarin medication interactions | (I1) Small group academic detailing & EMR alerts: 2 peer leaders delivered 40-minute sessions. A copy of key messages was mailed out 16 weeks after the sessions.  (C) EMR alerts only: drug-specific safety alerts describing the risk & recommending medication alternatives | Reduction in warfarin-interacting prescription rates at 18 months  (Academic detailing did not achieve additional effect, results pooled for analyses) | +22.4† |
| Simon S 2006 [32] | RCT & ITS  Drug safety | | 239 clinicians of a non-profit, group-model HMO in Oregon & Washington | To reduce inappropriate medication use in older people | (I1) Group academic detailing + computerized age-specific alerts: 2 "peer leaders" delivered the one-hour session at each clinic. A reminder letter mailed to each clinician.  (C) Computerized age-specific alerts describing the risk & recommending medication alternatives | Reduction in rate of targeted medication use  (Group detailing did not achieve additional effect, results pooled for analyses) | +5.7 |
| **iii. Reminders and telephone outreach** | | | | | | | |
| Bambauer K 2006 [33] | ITS  Depression | | 13,128 patients of a non-profit MCO in New England | Increase antidepressant medication adherence | A letter faxed to the prescriber if patients did not refill their antidepressant prescription for >10 days beyond the expected date | Reduction in % non-adherent patients | +2 with a trend of -0.3 per month |
|  |  |  |  |  |  | Reduction in % days without treatment | +2 with a trend of -0.4* per month |
| Raebel M 2005 [34] | RCT  Drug safety | | 10,169 patients of group-model HMO | Increase laboratory monitoring at initiation of drug therapy. Drugs included in the study are those with FDA black box warnings | Computerized alerts about incomplete laboratory test are sent to pharmacists daily. Pharmacists contact the patients by telephone to remind or order the test. | At 16 months:  Increase in dispensing with recommended laboratory monitoring completed | +8.9‡ |
| Raebel M 2006 [35] | RCT  Drug safety (selected 14 drugs) | | 9,139 patients of group-model HMO | Increase laboratory monitoring during ongoing therapy | See Raebel M 2005 | At 12 months:  Increase in patient-drug combinations with monitoring completed in the appropriate time frame | +6‡ |
| Palen T 2006 [36] | RCT  Drug safety | | 207 PCPs of 16 clinical facilities of a group-model MCO | Increase physician’s compliance with guidelines for laboratory monitoring at initiation of therapy | Non-intrusive alerts to physicians presenting information about laboratory monitoring for selected medications on the computer screens but do not require specific actions. | At 12 months:  Increase in dispensing with recommended laboratory monitoring completed | -0.5 |
| Feldstein A 2006 [37] | RCT  Drug safety | | 961 patients of 15 primary care clinics of a not-for-profit, group-model HMO | Increase laboratory monitoring at initiation of drug therapy | All interventions consisted of a reminder at baseline & again at ~10 days  (I1) Patient-specific EMR to the PCPs about missing laboratory test with sample letters for patients  (I2) Automated telephone voice message to the patient  (I3) A telephone call from a nurse to the patient to encourage testing. A follow-up letter sent to the patient | At ~2-weeks after second reminder: Increase in % patients completed laboratory test | (I1) +26.1*  (I2) +43.9*  (I3) +59.6* |
| Rickles N 2005 [38] | RCT  Depression | | 60 patients of 8 community pharmacies within a large MCO | Increase patient feedback, antidepressant adherence, medication knowledge, & improve depression symptoms. | Pharmacists: 3 monthly telephone calls providing education and monitoring - include recommendations on the management of side effects, referral to prescriber, & recommend discussion with a prescriber to select alternative medications with better adverse effect profiles or lower costs. | Improvement in antidepressant knowledge score | +23.3* |
|  |  |  |  |  |  | % Patients with improvement in depression symptom score at 3-months | +9.4 |
|  |  |  |  |  |  | Reduction in % patients missed doses at 6 months (not using intent-to-treat analysis) | +18.3* |
| Rickles N 2006 [39] | RCT  Depression | | See Rickles 2005 | See Rickles 2005 | See Rickles 2005 | Increase in patient feedback score (patient feedback to pharmacist) | +109‡ |
| Vollmer W 2006 [40] | RCT  Asthma | | 6,948 patients of a group-model HMO in Portland, Oregon | Improve quality of life & reduce acute healthcare utilization | Patients received 3 outreach calls (~5 months apart) providing supportive information over 10 months. Tailored feedback provided on asthma control & medication use based on the patient’s current asthma control; & alert provider for potential follow-up contact. | At 12 months:  Increase in % inhaled corticosteroids use, > 6 canisters | +0.6 |
|  |  |  |  |  |  | Reduction in % short acting beta-agonists use, > 6 canisters | +0.2 |
|  |  |  |  |  |  | Reduction in % emergency care or hospitalization | -0.1 |
| **III. FORMULARY INTERVENTIONS** | | | | | | | |
| **i. Tiered formulary** | | | | | | | |
| Huskamp H 2003 [42] | PP&C  ACE inhibitors, PPI, statins | 25,074 patients taking ACE inhibitors, PPI, or statins, of a large health plan | | Assess the effects of 2 different incentive-based formularies. | (I1) 1-tier formulary switching to 3-tier formulary & increasing the copayments for all tiers  (I2) 2-tier formulary switching to 3-tier & increases in the copayments for tier-3 drugs  (C1) & (C2) 2-tier formulary, matching based on similarity with regard to: the type of medical benefits, the copayment levels for 1- and 2-tiers, age, sex, & geographical distribution. | At 1 year:  Enrollees spending | (I1) +141.8^‡^  (I2) +7.5 |
|  |  |  |  |  |  | Plan spending | (I1) -58.2^‡^  (I2) -5.6 |
|  |  |  |  |  |  | % Patients continued use of tier-3 drug | (I1) -47.1^‡^  (I2) -18.8^‡^ |
|  |  |  |  |  |  | % Patients switched to drug of lower tier | (I1) +37.4^‡^  (I2) +26.1^‡^ |
|  |  |  |  |  |  | % Patients discontinued use of all drugs in class  (only analyses for ACE inhibitors presented here) | (I1) +9.8  (I2) -7.5 |
| Huskamp H 2005 [43] | PP&C  ADHD | 1,369 children, using ADHD medications, of a MCO offering PPO & point-of-service plans | | Adoption of a 3-tier formulary to attenuate increases in drug spending. Study to assess the effect of a 3-tier formulary adoption on use & spending patterns for ADHD medications for children. | (I1) 3-tier adoption: formulary changing from a 1-tier to a 3-tier structure  (I2) 3 drugs in tier-3 were moved to tier-2  (C) Comparison for I1 only: a plan that has a 2-tier formulary with stable cost-sharing | % Patients stayed with a medication of the same tier at 6 months | (I1) -5  (I2) 0 |
|  |  |  |  |  |  | Plan spending for ADHD patients | (I1) -43‡  (I2) +17‡ |
|  |  |  |  |  |  | Enrollees spending | (I1) +46‡  (I2) -7† |
| Motheral M 2001 [44] | PP&C | 20,160 patients of a PPO in the Midwestern US | | Assess the effect of a 3-tiered pharmacy benefit on pharmaceutical and medical utilization, & expenditures. | (I1) A 2-tier plan switched to a 3-tier plan  (C) a plan remained a 2-tier structure | At 12 months:  Prescription claims | -22.4‡ in tier-3; -4.4† in tier-2 |
|  |  |  |  |  |  | Member copayment | +34.4‡ |
|  |  |  |  |  |  | Net insurer costs | -21.8‡ |
|  |  |  |  |  |  | Office visits | 0 |
| Fairman K 2003 [45] | PP&C | 7,709 patients of a PPO in the Midwestern US | | See Motheral M 2001 | See Motheral M 2001 | At 2 years:  Prescription claims | -30.5† in tier-3; +2.2 in tier-2 |
|  |  |  |  |  |  | Member copayment | +46.5‡ |
|  |  |  |  |  |  | Net insurer costs | -26.7‡ |
|  |  |  |  |  |  | Office visits | +1.9 |
| Nair K 2003 [46] | PP&C | 8,132 patients of a managed care plan that included HMO, PPO, & Medicare+choice members | | Assess the impact of 3-tier (copayment) pharmacy benefit structures on medication use. | (I1) 2-tier copayment moving to 3-tier structure  (C1) 2-tier staying in 2-tier structure  (C2) 3-tier staying in 3-tier structure | Prescription per patient per month | +29 |
|  |  |  |  |  |  | Monthly copayment per member | +398.7 |
|  |  |  |  |  |  | Plan cost (per member) | +15.9 |
|  |  |  |  |  |  | Increase in formulary compliance rates | +6  (Effect sizes of I1 are calculated in comparison to C1) |
| Roblin D 2005 [47] | TS&C  Type 2 diabetes | 26,220 episodes of oral hypoglycemic use of 5 MCOs - covered 4 geographic areas of the US | | Medication cost-sharing to sensitize patients to the costs of medications, and attenuate increasing prescription drug use and costs. Study to assess effects of different levels of cost sharing on oral hypoglycemic use | (I1) large increase (> $10 per 30-day supply) in cost sharing  (I2) moderate ($7-$10) increase in cost sharing  (I3) small ($1-$6) increase in cost sharing | Change in trend in average daily dose of oral hypoglycemic per month at 12 months | (I1) -4.8†  (I2) -3.8  (I3) 0 |
|  |  |  |  |  |  | Observed average daily dose of oral hypoglycemic at 6 months compared with projections | (I1) -18.5  (I2) -9.2 |
| **ii. Prior authorization** | | | | | | | |
| Hartung D 2004 [49] | ITS&C  Arthritis | | 16,304 patients of Medicaid MCO, a not-for-profit MCO in the Portland, Oregon | Assess effects of a PA policy for celecoxib on pharmacy & medical service utilization. | A MCO instituted a PA policy for celecoxib. The PA policy required that patients had a diagnosis of osteoarthritis, rheumatoid arthritis or other chronic pain condition, plus any one of: history of GI bleeding, peptic ulcer, or corticosteroid therapy, age >60 years, or therapeutic failure to contraindication to >2 generic NSAIDs. | At 1 year, among patients:  % Celecoxib utilization | -48.5^‡^ |
|  |  |  |  |  |  | % Utilization of GI protectant | -8.1 |
|  |  |  |  |  |  | % Office visits | +2.3 |
|  |  |  |  |  |  | Monthly expenditure among enrollees on GI protectants  (only analyses for patients with a history of NSAIDs use presented here) | +8 |
| Gleason P, 2005 [50] | PP&C  Arthritis | | 737 patients of an employer group through a health plan in the Midwest | Assess the effects of a COX-2 inhibitor PA program on direct medical and pharmacy costs. | PA restriction for the COX-2 inhibitors celecoxib, rofecoxib, and valdecoxib. Members must meet the high GI event risk eligibility criteria. If the criteria were not met, physicians could submit a PA request to be medically reviewed. | For patients denied COX-2 inhibitor at 12 months:  Medical utilization per member | +11.5‡ |
|  |  |  |  |  |  | Pharmacy utilization per member | -31.3‡ |
| **iii. Formulary / coverage change** | | | | | | | |
| Gurwitz J 1995 [51] | ITS  Vaginal antifungal products | | 67, 365 females aged 11 years & over from a group model HMO | Examine impact of OTC availability of vaginal antifungal products on prescribing patterns and use of physician services | Availability of OTC anti-vaginal products | Antifungal prescriptions/month/100 members | -55 |
|  |  |  |  |  |  | Physician visits for vaginitis/month/100 members | +20 |
| McDonough K 1992 [52] | ITS  ACE inhibitors | | Patients taking enalapril in a staff-model HMO | Evaluate the impact of a voluntary formulary switch program | Patients taking enalapril asked by pharmacists if willing to switch to lisinopril. HMO agreed to waive copay for three months | Enalapril utilization (prescriptions/month) | -56 |
|  |  |  |  |  |  | Lisinopril utilization  (prescriptions/month) | +58 |
| Andrade S  2000 [53] | ITS  HRT therapy | | 7,778 patients dispensed HRT from a mixed-model HMO | Evaluate the impact of a formulary switch from conjugated to esterified estrogen tablets | Formulary switch from conjugated to esterified estrogen tablets | Number of esterified estrogen dispensing/month | -660 |
|  |  |  |  |  |  | Number of conjugated estrogen dispensing/month | 99 |
| Sullivan P 2005 [54] | PP&C  Allergic rhinitis (Prescription-to-OTC switch of loratadine) | | 58,329 patients. Plan sponsors include employers, unions, insurance and MCOs, their-party administrators, & state and federal employee programs | Assess effects of the prescription-to-OTC switch of loratadine & of different pharmacy benefit structures on drug utilization and cost. | Availability of OTC loratidine plus:  (I1) No change in formulary status of second generation antihistamines  (I2) All second generation antihistamines to third-tier  (I3) PA policy imposed for second generation antihistamines | At 12 months:  Prescriptions for second generation antihistamines | (I1) -66‡  (I2) -65‡  (I3) -88‡ |
|  |  |  |  |  |  | Prescriptions for all allergic rhinitis medicines | (I1) -88‡  (I2) -82‡  (I3) -95‡ |
|  |  |  |  |  |  | Pharmacy cost for all allergic rhinitis medicines | (I1) -146‡  (I2) -133‡  (I3) -125‡ |
| Delate T 2005 [56] | RCT  Drug adherence | | 6,518 patients of a Midwest-based health insurer - a variety of plans including PPO & HMO | Increase drug formulary adherence (switching to a formulary drug) | A notification letter to patients describing formulary change, the therapeutic options, the potential to lower out-of-pocket costs if switched to a formulary drug, & instructions to discuss with the prescriber about switching | Formulary adherence at 110 days | +33‡ |
| Delate T 2004 [55] | RCT  Drug dose optimization | | 1,521 prescribers of Health plans including PPO & indemnity plans | Optimize drug dose to convert the inefficient regimen to an equivalent daily dosage of the same drug or a different drug within the same therapeutic class | A pharmacy benefit manager with a health insurer encouraged a dose consolidation (dose optimization) program for financial savings.  (I1) Personalized letters to prescribers with information on their patients’ inefficient regimens & suggested dose consolidation options  (I2) Physician letter + patient letter: patients received a letter about dose consolidation describing the potential benefits, suggested dose consolidation options, suggestion to discuss with their prescribers | % Drug regimen changed to suggested efficient regimen at 180 days | (I1) +3.4  (I2) +6.3 |
|  |  |  |  |  |  | Change in pharmacy expenditure, per member per month | (I1) -$0.03  (I2) -$0.07 |
| DeZearn K 1996 [57] | PP&C  H2B | | Pharmacy benefit management company aiming to reduce costs in selected MCOs | Evaluate a program to promote generic cimetidine as the preferred H2B | Letter sent to prescribers about their patients on H2B therapy requesting change to generic cimetidine & asking to consider cimetidine for similar patients in the future. Also follow-up call 2 weeks after mailing to confirm receipt of letter | At 6 months:  % of brand cimetidine prescriptions | -5 |
|  |  |  |  |  |  | % of generic cimetidine prescriptions | 22 |
|  |  |  |  |  |  | % of ranitidine prescriptions | -21 |
| **IV. COLLABORATIVE CARE INVOLVING PHARMACISTS** | | | | | | | |
| Finley P 2003 [59] | RCT  Depression | | 125 patients of a staff-model, not-for-profit HMO in California | Improve drug adherence rates, patient outcomes, & examine associated medical resource utilization. | Pharmacists, with supervision of a psychiatrist, - titrate antidepressants, prescribe ancillary drugs (e.g. for sleep), follow-up via phone to assess drug adherence, & contact provider if changes were warranted | At 6 months:  Increase in drug adherence (MPR) | +7.8 |
|  |  |  |  |  |  | Change in resource utilization (primary care, emergency, psychiatric services visits) | -19 |
|  |  |  |  |  |  | Rates of benzodiazepine therapy | -8 |
| Okamoto M 2001 [60] | RCT  Hypertension | | 330 patients of a hypertension clinic within a MCO | Improve blood pressure control and patients’ perceived quality of care, & assess associated costs. | Pharmacists - provide education, reduce the number of antihypertensive drugs or alter therapy by administering more appropriate or less expensive drugs to achieve similar or improved blood pressure control, & order laboratory tests as needed; any changes conducted with physicians consent. | At 6 months:  Reduction in systolic blood pressure | +5.4^‡^ |
|  |  |  |  |  |  | Increase in quality of life: physical functioning in SF-36 | +5.6 |
|  |  |  |  |  |  | Total cost (drugs & visits) per patient | +4 |
| Rehring T 2006 [61] | PP&C  Peripheral arterial disease | | 691 patients of a group-model, not-for-profit MCO | Improve lipid control in patients with peripheral arterial disease. | Pharmacists - interact with patients & physicians. Pharmacists may recommend, initiate or titrate medications; & monitor medication and laboratory compliance. | At 6 months:  Increase in % patients received screening | +30‡ |
|  |  |  |  |  |  | Reduction in cholesterol level | +11‡ |
|  |  |  |  |  |  | Reduction in LDL level | +18.8‡ |
| Straka R 2005 [62] | PP&C  Hypercholesterolemia control | | 481 patients of 4 clinics of a 19-clinic staff model HMO in Minnesota | Improve management of hypercholesterolemia in patients with coronary heart disease whose LDL levels were not at goal. | Pharmacists with PCPs developed a patient-specific care plan to optimize hypercholesterolemia. Pharmacists - setting up & evaluating all initial and subsequent fasting lipid panels; titrating dosage when appropriate, & communicating with the responsible physician by email, phone, or face-to-face meetings. Pharmacists also educated patients about their new or adjusted dosage of lipid-lowering drugs & provided life-style advice. Patients were contacted by pharmacists via phone to follow-up fasting lipid panels and liver function tests, to notify them of changes in drug therapy or dosage, & for further education. | Reduction in LDL level at 6.5 months | +20.8‡ |
|  |  |  |  |  |  | Increase in % patients achieved the LDL goal level of less than 100 mg/dl at 6.5 months | +54‡ |
|  |  |  |  |  |  | Reduction in LDL level at 18 months after discontinuation of intervention | +12.2‡ |
| Borenstein J 2003 [63] | RCT  Hypertension | | 197 patients of a staff-model medical group affiliated with a community hospital | Reducing blood pressure in patients with uncontrolled hypertension. | Clinical guideline was used by clinical pharmacists in individual & group educational sessions provided to physicians. Patients with uncontrolled hypertension were identified & physicians informed. Patients attended a hypertension clinic run by pharmacists, patients’ blood pressures were determined, & patient assessment about medication adherence was collected. Pharmacists provided individualized patient education. Pharmacists provided assessment results & recommendations to PCPs who made final treatment decisions. | Reduction in systolic blood pressure | +6.5† |
|  |  |  |  |  |  | Increase in % patients with blood pressure goal achieved | +17* |
|  |  |  |  |  |  | Increase in % patients receiving at least one medication according to guideline | +2 |
|  |  |  |  |  |  | Total provider visits (physician & pharmacist visits) | +21.2 |
| **V. DISEASE MANAGEMENT INTERVENTIONS** | | | | | | | |
| Katon W 2002 [67] | RCT  Depression | | 228 patients of a HMO | Improve drug adherence, depressive symptoms, & assess health care costs. | Patients received educational book & video tape, 2 sessions with a psychiatrist, additional 2 sessions if necessary. Psychiatrists reviewed the current depressive episode & psychosocial history, may alter the dosage or choose an alternative medication. PCPs received immediate verbal consultation about patient progress & a written note within 1 week. | Increase in antidepressant adherence in severe patients at 12 months | +33* |
|  |  |  |  |  |  | Improvement in depression symptom score among ‘high severity’ subgroup | -2.5 |
|  |  |  |  |  |  | Unadjusted total health services costs | -6.2 |
| Katon W  2004 [68] | RCT  Depression | | 329 patients with both depression and diabetes mellitus of 9 primary care clinics from a non-profit HMO | Improve quality of care and outcomes of depression, & test intervention impact on diabetes outcome | The Pathways case management intervention provided enhanced education & support. Patients were initiated on antidepressant or problem-solving therapy followed by individualized, stepped-care depression treatment program provided by specialist nurses in collaboration with PCPs, i.e. patients received different types & intensities of services tailored to their observed outcomes. | Adequate antidepressant dosage at 12 months | +14.8 |
|  |  |  |  |  |  | Increase in drug adherence at 12 months | +11.2 |
|  |  |  |  |  |  | % Patient >50% improvement in depression symptom score | +9.4 |
|  |  |  |  |  |  | HbA1c level | +0.1 |
| Unutzer J 2001 [69] | RCT  Depression | | 1,356 patients of 46 primary care practices from 6 nonacademic MCOs in 5 states | Improve evidence-based management of depression, increase appropriate use of antidepressant medications and/or psychotherapy. | (I1) QI-therapy: referral to study-trained psychotherapists for cognitive-behavioral therapy  (I2) QI-medications: nurse specialists follow up patients taking antidepressant for up to 12 months to enhance adherence to guidelines for appropriate use of antidepressant medications | % Patients used antidepressants at 24 months | (I1) -6.8  (I2) +5.6 |
|  |  |  |  |  |  | % Patients used antidepressant or psychotherapy at 24 months | (I1) -5.6  (I2) +5.6 |
|  |  |  |  |  |  | Increase in antidepressant use according to guideline-recommended doses among patients with high risk for relapse (at 18 months) | (I1) -6.7  (I2) +12.4† |
| Ray W 2003 [70] | PP&C  Mental illness | | 8,151 patients – members of the Tennessee Medicaid program or 12 MCOs | Continue anti-psychotic therapy after change in mental health services coverage. | Transition of patients from traditional Medicaid and MCOs to specialty behavioral health organizations, i.e. a “carve-out” program. | At 12 months:  Improvement in % patients missed >60 days of anti-psychotic therapy | -2‡ |
|  |  |  |  |  |  | Improvement in % high-risk patients missed >60 days of therapy | -9.1‡ |
|  |  |  |  |  |  | Hospitalization for mental illness | +6.7 |
| Lozano P 2004 [71] | RCT  Asthma | | 638 patients of 42 primary care pediatric practices affiliated with 4 MCOs | Improve asthma care | (I1) Physician peer leader education: "peer leader" shares guidelines with colleagues & encourage implementation. Peer leaders received physician-specific feedback on anti-inflammatory prescribing by their colleagues.  (I2) Physician education program + planned care by nurses. Involve planned visits with assessments of asthma symptoms, medication use, care planning, & self-management support in collaboration with physicians. Nurses provided telephone follow-up between visits. Nurses reviewed quarterly reports on medication use & emergency visits with physicians. | At 2 years:  Reduction in annualized asthma symptom days in the past 14 days | (I1) +43.9  (I2) +89.9* |
|  |  |  |  |  |  | Reduction in use of oral steroids | (I1) +36†  (I2) +39† |
| Finkelstein J 2005 [72] | RCT  Asthma | | 1,796 patients with persistent asthma & 204 providers of 40 primary care practices affiliated with managed healthcare plans | See Lozano P 2004 | See Lozano P 2004 | At 2 years:  Change in patients dispensed >1 controller | (I1) +3  (I2) +4 |
|  |  |  |  |  |  | Change in patients dispensed >1 oral steroid | (I1) +6  (I2) +7 |
|  |  |  |  |  |  | Change in ambulatory visits | (I1) +6  (I2) +8 |
| Allison J 2003 [75] | RCT  H pylori infection | | 650 patients of a group-model HMO in Northern California | Decrease peptic ulcer disease symptoms & acid-peptic-related health care costs. | (I) Patients tested for HP and treated with triple therapy if they tested positive. Instructions about medication regimen provided. Patients re-tested 4 weeks after therapy. HP-positive patients were treated with quadruple therapy. HP-negative patients discontinued their acid-reducing medications.  (C) Continued acid suppression therapy | Reduction in gastrointestinal symptoms | +8.8* |
|  |  |  |  |  |  | Reduction in use of acid-reducing medication | +13.4‡ |
|  |  |  |  |  |  | Reduction in acid-peptic-related costs at 12 months | -44.8‡ |
| Ofman J 2003[76] | RCT  H pylori infection | | 406 patients of a group-model MCO | Increase compliance with HP testing, use of appropriate anti-HP therapy, & examine economic outcomes. | Test-and-treat guidelines were developed locally and implemented. Nurses performed HP testing. Guidelines implemented via a single group meeting with a local physician champion, academic detailing by pharmacists with 3 follow-up group meetings, & nursing and pharmacist education. Pharmacists provided patient education. Nurses monitored patients via phone & encouraged compliance. | Increase in compliance with HP testing | +52‡ |
|  |  |  |  |  |  | % Patients prescribed appropriate anti-HP therapy | +86‡ |
|  |  |  |  |  |  | Total costs at 6 months | -6.6 |

Abbreviations

Design: PP= pre-post; &C= with comparison group; RCT= randomized control trial; TS= time series; ITS= interrupted time series

Goal/s, Intervention/s, Key Outcomes: ACE inhibitors= angiotensin-converting enzyme inhibitors; ADHD= attention deficit hyperactivity disorder; BMD= bone mineral density; C= comparison group; CDC= Center for Disease Control; EMR= electronic medical record; FDA=Food and Drug Administration; GI= gastrointestinal; H2B= histamine-2 blockers; HMO=health maintenance organization; HP= Helicobactor pylori; I1= intervention 1; I2= intervention 2; IPA=independent practice association; LDL= low-density lipoprotein; MCO=managed care organization; MPR=medication possession ratio; NSAIDs= non-steroid anti-inflammatory drugs; OTC=over-the-counter; PA= prior authorization; PCP= primary care physician; PPI= proton pump inhibitors; QI=quality improvement; TCA= tricyclic-antidepressants.

**^δ^**The procedure for calculating effect size depended on the outcome in question. If the outcome was measured as a percentage, effect size was computed as the relative gain in the intervention group, that is, the net difference between the percent improvement in the intervention group and the net difference in the control group [(%POST-%PRE)_intervention_ -(%POST-%PRE)_control_]. If the outcome was a rate or a score, the changes (i.e. improvements pre to post) were converted to percent improvements by dividing the absolute changes by baseline values [((POST-PRE)/PRE)_intervention_ -((POST-PRE)/PRE)_control_]. In the methodologically acceptable studies without a control group (i.e. time series/repeated measures), effect size was calculated as percent improvement between the pre-intervention percentage and the post intervention percentage; short-term shifts in percentage immediately before or after an intervention were regarded as transitory.

* p<0.05

^†^ p<0.01

^‡^ p<0.001
